# Videolaryngoscopy in critically ill patients

**DOI:** 10.1186/s13054-019-2487-5

**Published:** 2019-06-17

**Authors:** Samir Jaber, Audrey De Jong, Paolo Pelosi, Luca Cabrini, Jean Reignier, Jean Baptiste Lascarrou

**Affiliations:** 1 PhyMedExp, University of Montpellier, INSERM U1046, CNRS UMR 9214, Montpellier, France; 2 Anesthesia and Critical Care Department B, Saint Eloi Teaching Hospital, Centre Hospitalier Universitaire Montpellier, 34295 Montpellier Cedex 5, France; 3 Department of Surgical Sciences and Integrated Diagnostics, University of Genoa, Genoa, Italy; 4 San Martino Policlinico Hospital, IRCCS for Oncology and Neurosciences, Largo Rosanna Benzi 8, 16131 Genoa, Italy; 5 Department of Anaesthesia and Intensive Care, IRCCS San Raffaele Scientific Institute, Via Olgettina 60, 20132 Milan, Italy; 6 Università Vita-Salute San Raffaele, Via Olgettina 58, 20132 Milan, Italy; 7 Medicine Intensive Reanimation, University Hospital, Nantes, France

**Keywords:** Videolaryngoscopy, Direct laryngoscopy, Tracheal intubation, Critically ill, Emergency department, Intensive care unit

## Abstract

Intubation is frequently required for patients in the intensive care unit (ICU) but is associated with high morbidity and mortality mainly in emergency procedures and in the presence of severe organ failures. Improving the intubation procedure is a major goal for all ICU physicians worldwide, and videolaryngoscopy may play a relevant role.

Videolaryngoscopes are a heterogeneous entity, including Macintosh blade-shaped optical laryngoscopes, anatomically shaped blade without a tube guide and anatomically shaped blade with a tube guide, which might have theoretical benefits and pitfalls. Videolaryngoscope/videolaryngoscopy improves glottis view and allows supervision by an expert during the intubation process; however, randomized controlled trials in the ICU suggest that the systematic use of videolaryngoscopes for every intubation cannot yet be recommended, especially in non-expert hands. Nevertheless, a videolaryngoscope should be available in all ICUs as a powerful tool to rescue difficult intubation or unsuccessful first-pass laryngoscopy, especially in expert hands.

The use of associated devices such as bougie or stylet, glottis view needed (full vs incomplete) and patient position during intubation (ramped, sniffed position) should be further evaluated. Future trials will better define the role of videolaryngoscopy in ICU.

## Introduction

Patients in the intensive care unit (ICU) frequently require endotracheal intubation, which is associated with high morbidity and mortality mainly during emergency procedures and the presence of severe cardiorespiratory insufficiency. Thus, in these conditions, it is extremely important to improve both the intubation procedure as well as safety, and videolaryngoscopy may play a relevant role as compared to conventional intubation techniques. Different videolaryngoscopes are currently available, including Macintosh blade-shaped optical laryngoscopes (traditional geometry), anatomically shaped blade (hyperangulated) without a tube guide and anatomically shaped blade (hyperangulated) with a tube guide, which might have theoretical benefits as well as possible pitfalls. In the present paper, different points of view regarding the use of videolaryngoscopy compared to conventional intubation in critically ill patients will be discussed taking into consideration possible benefits, negative aspects and potential contraindications, in different clinical conditions, according to current scientific evidence. This information may be helpful to the clinicians to better identify patients at risk of difficulties during intubation, choose the optimal technique and possibly avoid life-threatening complications, thus likely improving clinical outcome.

## Samir Jaber and Audrey De Jong: videolaryngoscopy in critically ill patients—yes

Videolaryngoscopes (VLs) are proposed to improve airway management and to reduce incidence of difficult intubation in the operating room. These devices contain a miniaturized camera aimed at the tip of the blade to indirectly visualize the glottis. Although VLs have rapidly become routine devices for airway management in many situations, the use in an intensive care unit (ICU) surrounding is more recent than in operating rooms and the effectiveness of VLs in increasing first-attempt success and reducing difficult intubation or complications related to intubation remains debated [1].

VLs are not a homogeneous class. Table 1 presents the main characteristics of the VL available on the market and respective advantages and inconveniences. They differ in design, technical configuration and blade type, requiring the user to become familiar with each device before use in a difficult airway situation. There are 3 main categories of VLs according to the type of blade: (1) Macintosh blade-shaped optical laryngoscopes (traditional geometry): These devices have Macintosh blades but are combined with video technology. The glottis can be seen either directly or on a video screen. (2) Anatomically shaped blade (hyperangulated) without a tube guide: The blade is anatomically shaped (hyperangulated), giving a view of the glottis without the need to flex or extend the neck. These VLs provide only an indirect view of the glottis, and a preshaped stylet needs to be placed into the endotracheal intubation tube (EIT) before intubation. (3) Anatomically shaped blade (hyperangulated) with a tube guide: A preshaped stylet is not needed.

**Table 1:** Classification of videolaryngoscopes available in ICU and respective advantages and inconveniences

Videolaryngoscopes (VLs)	Advantages	Inconveniences
VL without channel (example: Glidescope (Verathon), Mc Grath serie 5 (Medtronic/Covidien), C-mac D-blade (Karl Storz), Kingvision non channelled (Ambu) etc.)	- Angulated blade (improve glottis view of + 2 Cormack)	- Use of stylet mandatory to pre-shape the endotracheal tube - Difficulty to enter the tube into the trachea through the glottis (importance of training)
VL with channel (example: Airtraq (Vygon), Airway scope (Pentax), Kingvision channelled (Ambu) etc.)	- Angulated blade with channel (improve glottis view of + 2 Cormack) - No need of stylet (the tracheal tube is introduced in the channel)	- Size of the device in case of limited opening mouth - Difficulty to enter the tube into the trachea through the glottis (importance of training)
Combo (or "Macintosh") VL (example: Mc Grath Mac (Medtronic/Covidien), APA (Care fusion), C-mac (Karl Storz) etc.)	- Direct and indirect laryngoscopy using the same standard Macintosh shaped-blade - Possibility to insert an angulated blade on the same device - With or without channel - With deported or included screen	- Indirect laryngoscopy with a standard Macintosh blade: improve glottis of + 1 Cormack (instead of + 2 Cormack with an angulated blade)
VL with deported screen (example: Glidescope (Verathon), C-mac (Karl Storz), APA (Care fusion) etc.)	- Large screen - Educational	- Cumbersome
VL with screen included on the device (example: C-mac pocket (Karl Storz), Mc Grath Mac (Medtronic/Covidien), APA (Care fusion), Airtraq (Vygon), Kingvision (Ambu) etc.)	- Portable	- Smaller screen - Less educational than a deported screen

One VL can belong to several categories. *VLs* videolaryngoscopes

In the past decade, the role of VLs has been discussed, particularly in ICU where scientific evidence was lacking and intubation conditions are more difficult than in the operating room [2]. In a before-after study reporting a quality improvement process using a VL in an airway management algorithm [3], the systematic use of a combo VL for intubation significantly reduced the incidence of difficult intubation and/or difficult laryngoscopy [3]. In the multivariate analysis, the “standard laryngoscopy” group was an independent risk factor for difficult intubation and/or difficult laryngoscopy, as was a Mallampati III or IV score and a non-expert operator status. In addition, in the subgroup of patients with difficult intubation predicted by the MACOCHA score [4], the incidence of difficult intubation was much higher in the “standard laryngoscopy” group (47%) than in the “combo VL” group (0%).

In 2014, a systematic review and meta-analysis [5] provided evidence that VL could be useful in airway management of ICU patients. Among seven evaluated outcomes of interest, in comparison to direct laryngoscopy, VL improved four of them (difficult intubation, first-attempt success, Cormack 3/4 grades, esophageal intubation) and did not modify three of them (severe hypoxemia, severe cardiovascular collapse, airway injury). Following these promising studies performed in critically ill patients [3, 5, 6], the use of VLs was recommended very early (first-line or after a first-attempt failure using direct laryngoscopy) in ICU airway management algorithms [7], including the British [8] and French [9] recommendations. However, the main challenge with the VL is more to insert the EIT into the trachea than visualizing the glottis. Achieving a 100% percentage of glottis opening (POGO) view (corresponding to a Cormack-Lehane grade 1 in direct laryngoscopy) during VL does not guarantee successful intubation. Progression of the EIT into the trachea is sometimes difficult because the EIT has to pass a sharp angle to enter the larynx. It is worth reminding that it is not the laryngoscopy that leads to the decompensation in these patients. It is more likely their physiologic disturbances. Avoiding the difficult airway will lead to less decompensation because of prolonged laryngoscopy. The current literature on RCTs does not address this and either directly or indirectly exclude patients with predicted difficult airways in every case except in the trial performed by Lacarrou et al. [10]. A large multicenter randomized controlled trial [10] performed in an ICU setting compared VL to DL regarding the rate of the first-attempt successful intubation and reported intriguing results that could be interpreted either way. First, as reported in the author’s conclusion, a combo VL compared to DL did not improve first-pass orotracheal intubation rates and was associated with higher rates of severe life-threatening complications. Second, it is worth noting that more than 80% of the operators were non-expert, without experience in the field of intubation, and in particular in the use of VL. Moreover, a stylet was used in less than 20% of attempts. Using traditional geometry VL as a VL, the tongue and upper airway are not compressed so a stylet is often needed. For us, the main message of the paper could be that when using any tool (here a VL) in non-trained and non-expert operators, this tool may be not efficient and even harmful. Following this study [10], meta-analyses [11, 12] were published. Although no difference was reported in first-attempt success between VL and DL, these non-significant results could be interpreted with caution because of the high heterogeneity between trials reaching 73%.

As reported in operating rooms [13], training and education are essential in order to improve patient safety during endotracheal intubation using a VL. Long-term training needs to be emphasized when new VL devices are introduced into practice, especially since intensivists perform intubation less frequently than anesthesiologists. The experience required to attain 90% probability of optimal performance with VL has been evaluated [13, 14]. At least 75 VL attempts were required to achieve that level of proficiency [13, 14]. Inexperienced operators tend to lose time when attempting EIT insertion under indirect vision, increasing time to intubate which is associated with increased intubation-related complications, such as hypoxemia. There is no universal ideal VL, and each type of device requires a learning curve. VLs should not be used as a first-line device for all intubations in non-experienced operators. However, the learning curve is steeper with VL than with direct laryngoscopy [15, 16]. One could argue that VL should be the go-to device given that trainees do not intubate as frequently as anesthesiologists, it is the preferred device in the face of difficulty and difficulty is quite unreliable to predict. Expertise must be acquired in non-difficult intubation first to reach optimal performance in difficult intubation. The choice of a videolaryngoscope should be based on difficult intubation situations and not just “classical” intubation. Finally, using VL may be of great help for an experienced operator in both non-difficult and difficult intubations in the ICU setting.

## Paolo Pelosi and Luca Cabrini: videolaryngoscopy in critically ill patients—maybe

In critically ill patients, difficult tracheal intubation is more common compared to elective surgical patients [17, 18]. Moreover, the risk of severe complications is higher than in non-critically ill patients and proportional to the number of intubation attempts [19, 20]; hence, it is relevant to improve the first-pass intubation rate, independently from operator experience. VLs are increasingly used in critically ill patients, to improve first-attempt intubation rate, to reduce intubation failure and intubation time and to reduce adverse events.

A recent comprehensive systematic review addressing tracheal intubation in the critically ill patients [21] identified nine randomized controlled trials (RCTs) comparing VL to traditional direct laryngoscopy patients. VL did not offer better results in terms of first-attempt intubation rate and intubation time independently from the level of experience of the operator, the setting (intensive care unit (ICU) vs emergency department), the presence or not of hyper-angulation of the blade of the VL and even the presence or not of anticipated difficult airway. Furthermore, in the two largest trials, VL appeared to be associated with an increased incidence of severe complications and deaths [10, 22]. These findings were confirmed in four subsequent studies. In a RCT including 163 ICU patients, the rate of first-pass intubation, intubation failure, number of attempts and complications were comparable between VL and direct laryngoscopy [23]. In a retrospective study on emergency intubation in general wards by medical emergency team, VL offered a better first-attempt success rate but no benefits in intubation-related complications; moreover, the incidence of severe desaturation and mortality were higher with VL [24].

In a meta-analysis on VL versus direct laryngoscopy in 15,064 emergency intubation outside the operating room (including non-randomized trials), no difference was found in first-pass intubation rate, even if subgroup analysis showed better results with VL in the ICU, in less experienced operators and when using the C-MAC (Karl Storz, Tuttingen, Germany); VL was associated with a greater incidence of arterial hypotension, but the overall rate of complications and the rate of esophageal intubation were lower [25]. Finally, in a RCT comparing an EIT with an integrated video camera versus direct laryngoscopy, no advantage was found [26].

Are we at the (un)happy end of the affair between VL and critically ill patients? Maybe, no. Despite the disappointing results reported above, there could still be a room for this device in critically ill patients. What have we learned? First, routine use of VL in unselected critically ill patients cannot be recommended: even if some studies observed that VL offers a better vision, this does not translate in better first-pass intubation rate or lower complication rate. However, given the high incidence of difficult airway in this population [8], and despite the negative results by Lascarrou et al. [10] in subgroup analysis, VL might be one rescue technique after failure to intubate using direct laryngoscopy. Accordingly, recent guidelines stated that “A videolaryngoscope should be available and considered as an option for all intubation of critically ill patient” [8]. Second, the fact that a better glottis view is not followed by better first-attempt intubation rate could imply that clinicians need better training on this device. Expert clinicians (that is, expert in the use of direct laryngoscopy) showed no benefit from using VL; on the contrary, clinicians with less experience (with no gap or with a reduced gap in experience between VL and direct laryngoscopy) showed better results when using VL [25]. Adequate training is of the essence [8]; simulation could be a valuable aid. Third, VL includes an increasing variety of quite different devices. So far, we have conflicting results about the superiority of one device over the others in critical settings [21, 25]: largest studies are required to identify the potentiality of single devices. Every videolaryngoscope has its features. Intensivists with a large experience with intubation have very different opinions on the different types of videolaryngoscopes, during difficult intubation. Some videolaryngoscopes make intubation more difficult depending on the place they take in the mouth of the patients with limited mouth opening, for example. If the choice is made on a type of videolaryngoscope that allows “simple” intubations in patients without difficult intubation criteria, non-experts can be falsely reassured by this tool. The chain of warning of difficult intubation criteria will not be triggered, with a lack of anticipation of difficult intubation algorithm. Fourth, we should be aware also of the limits and risks of VL. VL appears associated with severe adverse events as pulse-oximetry desaturation or arterial hypotension [10, 21], or even worse survival rate in some settings [22, 24]: better results might be obtained avoiding VL in patients with markedly labile respiratory or hemodynamic conditions, or when intubating general ward or head trauma patients. Moreover, future research could identify the risk factors for VL failure, like airway edema, cervical immobility, presence of blood in the airway and obesity [27].

In our opinion at present, VL should not be recommended for routine use in critically ill patients; moreover, VL could be detrimental in some conditions. Nevertheless, we still believe that it could be a valuable rescue technique with the right device and in trained hands facing failed attempt(s) with direct laryngoscopy. Randomized studies assessing VL versus other rescue techniques, performed by previously trained clinicians, are required before banning VL from critically ill setting. Please keep the door of the ICU still open to VL—and actually, it is already present in half of them! [28].

## Jean Reignier and Jean Baptiste Lascarrou: videolaryngoscopy in critically ill patients—no

“Technology is a word that describes something that doesn’t work yet.” Douglas Adams

Endotracheal intubation (ETI) of critically ill patients is very often required but is also associated with a high risk of complications [17]. Considerable research effort has therefore been expended to make ETI easier and safer in the ICU. One product of this research is the development of videolaryngoscopes designed to provide a better view of the larynx compared to direct laryngoscopes. The goals of videolaryngoscopy for ETI in the ICU are to increase the first-pass success rate and to shorten the time needed to achieve ETI, ensuring constant supervision and teaching by airway expert for all operators regardless of experience thereby improving patient outcomes. The use of videolaryngoscopy requires specific training supplied by an expert and constant supervision until expert status is achieved.

In ICU patients, videolaryngoscopy can be used either routinely for all ETI procedures or only in selected patients. International guidelines recommend the use of videolaryngoscopy when screening tests predict difficult intubation, ETI with direct laryngoscopy fails or the patient is critically ill [29, 18]. However, this recommendation is based chiefly on studies performed in an operating room, as opposed to clinically unstable patients in the ICU. Furthermore, reliably predicting the ease of ETI is difficult in the setting of emergency care provided to highly unstable patients. Finally, using videolaryngoscopy only after failure of ETI with direct laryngoscopy delays the institution of effective ventilatory assistance and may therefore jeopardize patient outcomes. A further obstacle to this last strategy is that it requires the availability of both a direct laryngoscope and a videolaryngoscope, as well as of operators trained in both methods [30].

The routine use of videolaryngoscopy for all ETIs in the ICU may therefore seem preferable. The rationale is that videolaryngoscopy, by providing a better view of the larynx in virtually all situations, should facilitate proper EIT placement, thereby increasing the success rate, shortening the ETI procedure and decreasing the risk of complications, even when performed by “non-experts” in unstable critically ill patients. A single-centre randomized controlled ICU trial showed that videolaryngoscopy provided a higher first-pass success rate of emergent ETI performed by clinical pulmonology or critical care fellows, with no difference in complication rates, compared to direct laryngoscopy [31]. However, in two other single-centre randomized controlled ICU trials, videolaryngoscopy improved glottis visualization but failed to increase the ETI success rate or to decrease the complication rate compared to direct laryngoscopy [32, 33]. In one of these trials [32, 33], videolaryngoscopy was associated with a lower median SpO_2_ during ETI, raising concern about an adverse impact on patient outcomes. Similarly, in the only multicentre randomized trial in critically ill patients, videolaryngoscopy not only failed to improve the first-pass success rate, but also was associated with a higher frequency of a composite endpoint of death, cardiac arrest, severe hypoxemia and arterial hypotension [10]. A 2017 meta-analysis included all 12 available randomized controlled trials of videolaryngoscopy versus direct laryngoscopy in critically ill patients [34]. The first-pass success rate was not significantly different for ETI performed in the hospital, regardless of operator experience, and was lower with videolaryngoscopy for ETI performed by experienced operators during the prehospital procedure. In sum, well-designed studies indicate that the better view of the glottis provided by videolaryngoscopy compared to direct laryngoscopy does not translate into a higher ETI success rate and that videolaryngoscopy may be associated with increased risks to critically ill patients.

Despite these findings, videolaryngoscopy is widely used. Thus, in a randomized trial reported in 2018 and comparing a bougie to an EIT with a stylet for the emergency ETI of patients with possible difficult airways, videolaryngoscopy was used in over 99% of patients [35]. The current challenge would therefore seem to lie in improving the outcomes of videolaryngoscopy. One difficulty with videolaryngoscopy is that the view may appear distorted, with complete glottis exposure but failure to visualize the EIT until it reaches the hypopharynx. Consequently, proper EIT placement with videolaryngoscopy may require the use of additional devices such as a stylet, bougie or channelled-blade videolaryngoscope. Also, the disappointing results obtained with videolaryngoscopy may be ascribable to insufficient operator training. The learning curves of direct laryngoscopy and videolaryngoscopy have not been compared directly. However, acquiring proficiency required at least 50 ETIs with direct laryngoscopy [36] and 76 ETIs with videolaryngoscopy [14]. A simulation training study suggests that training in direct laryngoscopy may help to acquire videolaryngoscopy skills, raising concern that providing only training in videolaryngoscopy might put patients at risk should an operator be forced by circumstances to use direct laryngoscopy [37]. Finally, no reliable comparison of the many available videolaryngoscopes has been published to date.

**Fig. 1:**
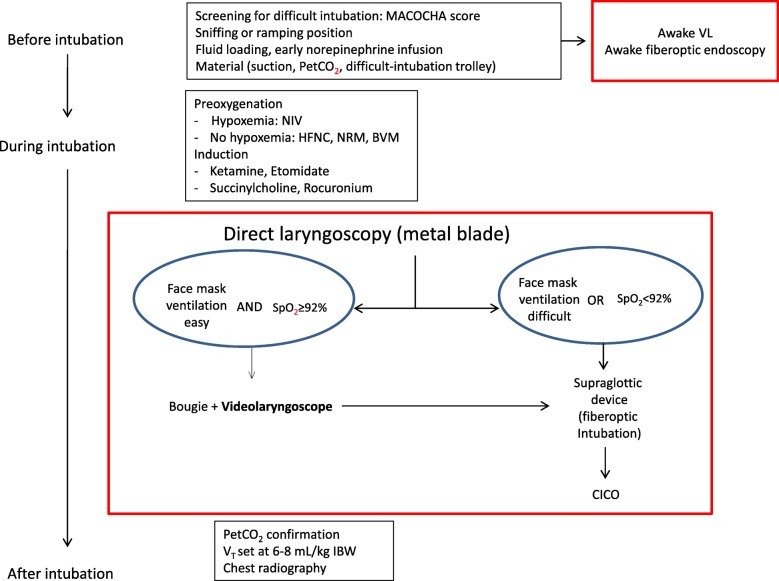
Videolaryngoscopy and intubation in critically ill patients

Clearly, further work is needed to define the technical videolaryngoscope characteristics capable of translating the better view of the glottis into higher first-pass success rates, as well as the training modalities that ensure optimal operator performance during ETI with videolaryngoscopy. However, videolaryngoscopy has already a place in the intubation process in the ICU and somewhere between difficult intubation and before hypoxemia as shown on Fig. 1.

Large studies with standardized operator training and patient-centred outcome measures are needed to compare various videolaryngoscopy modalities. The classification of ETI-related life-threatening complications suggested by Jaber et al. may assist in this endeavour. Videolaryngoscopy may well constitute an advance for emergency, critically ill and surgical patients in the future but should not yet be viewed as having fully earned its credentials. We must continue to learn about which technical characteristics make the best videolaryngoscope, which training methods produce the best operators and which patients are most likely to benefit.

## Conclusions

Videolaryngoscopes are a heterogeneous entity, improving glottis view and allowing supervision by an expert during the intubation process. In an ICU setting, videolaryngoscopes for every intubation cannot yet be recommended. Nevertheless, a videolaryngoscope should be available in all ICUs as a powerful tool to rescue difficult intubation or unsuccessful first-pass laryngoscopy, especially in expert hands. Future trials will better define the role of videolaryngoscopy in ICU.

## Data Availability

Not applicable.

## Abbreviations

ETI: Endotracheal intubation
ICU: Intensive care unit
MACOCHA: Mallampati score of III or IV, apnea syndrome (obstructive), cervical spine limitation, hypoxemia, opening mouth < 3 cm, coma and operator being a non-anesthesiologist (5 points for Mallampati score, 2 points for apnea and 1 point each for all others)
RCT: Randomized controlled trial
VL: Videolaryngoscope/videolaryngoscopy
